# Narrowing the Gap in Differential Attainment for Psychiatry Core Trainees in East Midlands Through Mentorship Scheme

**DOI:** 10.1192/bjo.2024.430

**Published:** 2024-08-01

**Authors:** Champion Seun-Fadipe, Sewanu Awhangansi, Samreen Samad, Oluwaseun Oluwaranti, Ian Yanson

**Affiliations:** 1 Nottinghamshire Healthcare NHS Trust, Nottingham, United Kingdom; 2 Leicestershire Partnership NHS Trust, Leicester, United Kingdom

## Abstract

**Aims:**

The use of mentorship schemes may be a pragmatic approach to bridging the differential attainment gap for psychiatry trainees. There is robust evidence that mentorship improves outcomes for core trainees across several domains including exam pass rates, ARCP outcomes and clinical practice. A survey was developed to elicit core psychiatry trainees’ perspective about the need for mentoring as well as their expectations. This was an initial survey done as part of a Quality Improvement project focused on mentoring scheme for psychiatry core trainees in the East Midlands region.

**Methods:**

A 16-item self-rated questionnaire was designed to elicit information relating to respondents’ demographics, professional qualifications, UK experience prior to commencement of training, perception of mentorship as an unmet need as well as expected focus of potential mentoring relationship. These were administered to psychiatry core trainees in the East Midlands region. The data was collected in February 2023.

**Results:**

About a quarter of the core trainees (n = 21) participated in the survey. Majority (47.6%) of the respondents had Black or Black British ethnic origin and 11 (52.4%) were in their second year of training. Although 13 (61.9%) had a non-UK primary medical qualification, majority had some months of UK experience before commencement of training (median = 1.4 years). Twenty (95%) of the respondents identified mentoring as an unmet need and they highlighted the areas of need.

**Conclusion:**

This survey showed a high level of acceptance of the mentoring scheme among the trainees. Their expectations and suggestions helped further the design of the mentoring scheme which is currently ongoing.

